# A dyadic analysis of family adaptation among breast cancer patients and their spouses based on the framework of family stress coping theory

**DOI:** 10.3389/fpubh.2024.1453830

**Published:** 2024-11-25

**Authors:** Zhangyi Ding, Yarong Fan, Gaoxiang Zhong, Xinmiao Zhang, Xichen Li, Yan Qiao, Huixia Cui

**Affiliations:** ^1^School of Nursing, Jinzhou Medical University, Jinzhou, Liaoning, China; ^2^School of Nursing, Shanxi Medical University, Taiyuan, Shanxi, China; ^3^School of Nursing, Wannan Medical College, Wuhu, Anhui, China

**Keywords:** dyadic coping, family adaptation, benefit finding, family stress coping theory, actor-partner

## Abstract

**Background:**

The active coping strategies of family members can help breast cancer patients better handle the crisis, and family adaptation is a manifestation of the family's active coping with the crisis. In the study of breast cancer, a disease that predominantly affects women, we explored the influence of spouses on patients' family adaptation. This aspect has not been explored in previous studies.

**Purpose:**

In recent years, with the development of family stress coping theory, cancer coping styles have shifted from an individual focus to a whole-family approach. This shift has the potential to help families of cancer patients adapt to the crisis. This study aimed to explore the correlation between dyadic coping, family adaptation, and benefit finding in couples with breast cancer.

**Methods:**

Using convenience sampling, the study included 325 pairs consisting of breast cancer patients and their spouses who attended breast surgery, oncology, and chemotherapy sessions between April and November 2023. The survey utilized the General Information Questionnaire for patients and spouses, the Dyadic Coping Scale, the Benefit Finding Scale, and the Family Adaptability and Cohesion Evaluation Scales. Data analysis was conducted using SPSS 25.0 and Amos 24.0 software.

**Results:**

In the actor effect of dyadic coping on family adaptation, the benefit finding of patients and their spouses played a mediating role. Regarding the partner effect (*B* = 0.019, 95% CI = 0.003–0.045, *P* < 0.05), the dyadic coping of spouses indirectly affected the family adaptation of patients through the benefit findings of patients. The patient's dyadic coping can directly affect the spouse's family adaptation. The spouse's dyadic coping can influence the patient's benefit finding.

**Conclusion:**

There is a partial interaction between breast cancer patients and their spouses' dyadic coping, benefit finding, and family adaptation. Therefore, clinical staff should promptly identify patients and spouses with poor coping abilities and provide them with positive psychological interventions to enhance the dyadic coping abilities of both partners and assist them in overcoming the problems encountered during the treatment process, ultimately helping them better cope with family crises.

## Introduction

According to the data released by the International Agency for Research on Cancer in 2020, the number of new cancer cases reached 19.3 million worldwide. Notably, breast cancer cases accounted for 2.3 million of these cases, surpassing lung cancer to become the most common cancer globally (1). In 2020, there were an estimated 4.82 million new cancer cases in China. Among these, the number of new cases of female breast cancer reached 357,200, making it the most common cancer among Chinese women after lung, rectal, thyroid, and liver cancers (2).

The diagnosis and treatment of cancer can often be extremely stressful for patients, resulting in severe negative emotions and other adverse consequences (3). More seriously, such stressful events may adversely affect the family adaptation of cancer patients (4). Previous research has shown that couples dealing with illness together can enhance their feelings of closeness and reduce the stress and psychological burden associated with the illness (5). In the face of crisis events, families of cancer patients adopt different coping styles, which may affect the family's adaptation (6).

Family adaptation is the family's direct response to a stressful event, indicating that the family needs to readjust its structure to restore stability and improve overall happiness and satisfaction (7). Studies have shown that stressful events reduce the family's capacity to adapt for both the patient and the primary caregiver (8). In the course of breast cancer treatment, the adaptive capacity of the patient's family is closely related to the caregiver's perceived stress; the higher the perceived level of stress, the worse the adaptive capacity of the family (9). Good family adaptation can reduce the incidence of suicidal ideation in patients (10), and the incidence of anxiety and depression is relatively low in patients with good family adaptation (11).

Benefit finding is a cognitive way of actively coping with adverse external circumstances, manifested as benefits finding from traumatic or unfortunate life events (12). In studies of cancer in men, it has been confirmed that benefit finding is a cognitive strategy to cope with cancer actively; the higher the level of benefit finding, the higher the level of quality of life (13). This cognitive approach not only reduces caregivers' negative emotions, such as anxiety and depression, improves mental health (14), but also improves overall quality of life (15). However, some studies have found that the extent of benefit finding may decrease as the patient's condition worsens (16). A study observed that the level of caregiver benefit findings was strongly correlated with family adaptation (17). In the dyadic study, it was noted that for older patients with physical impairments and their caregivers, low levels of benefit finding may produce mental and emotional distress, which in turn reduces the level of family adaptation (18). Therefore, it is necessary to study further the effect of benefit finding between couples on family adaptation.

In recent years, with the development of the family stress coping theory, the coping style of cancer has shifted from an individual focus to the couple's experience, that is, dyadic coping (19). Research has shown that couples who adopt good dyadic coping strategies can regulate their stress levels more effectively and navigate difficult situations with ease (20). This ability to cope constructively can enhance mutual communication and dependence between couples, foster a more intimate relationship, and improve their ability to resist the illness together (21). Families with better coping styles are more capable of overcoming difficulties and increasing their confidence in dealing with the disease (22). In a dyadic study, it was found that couples facing breast cancer exhibited inefficient coping styles, which not only causes serious psychological distress for both spouses but also may lead to a coping crisis within the family (23). A good dyadic coping style between a husband and wife not only helps effectively manage the disease and overcome the illness but also contributes to a more stable and harmonious family environment (24). Therefore, whether the dyadic coping style between couples has a positive or negative impact on family adaptation needs further research.

## Conceptual framework

**Figure 1:**
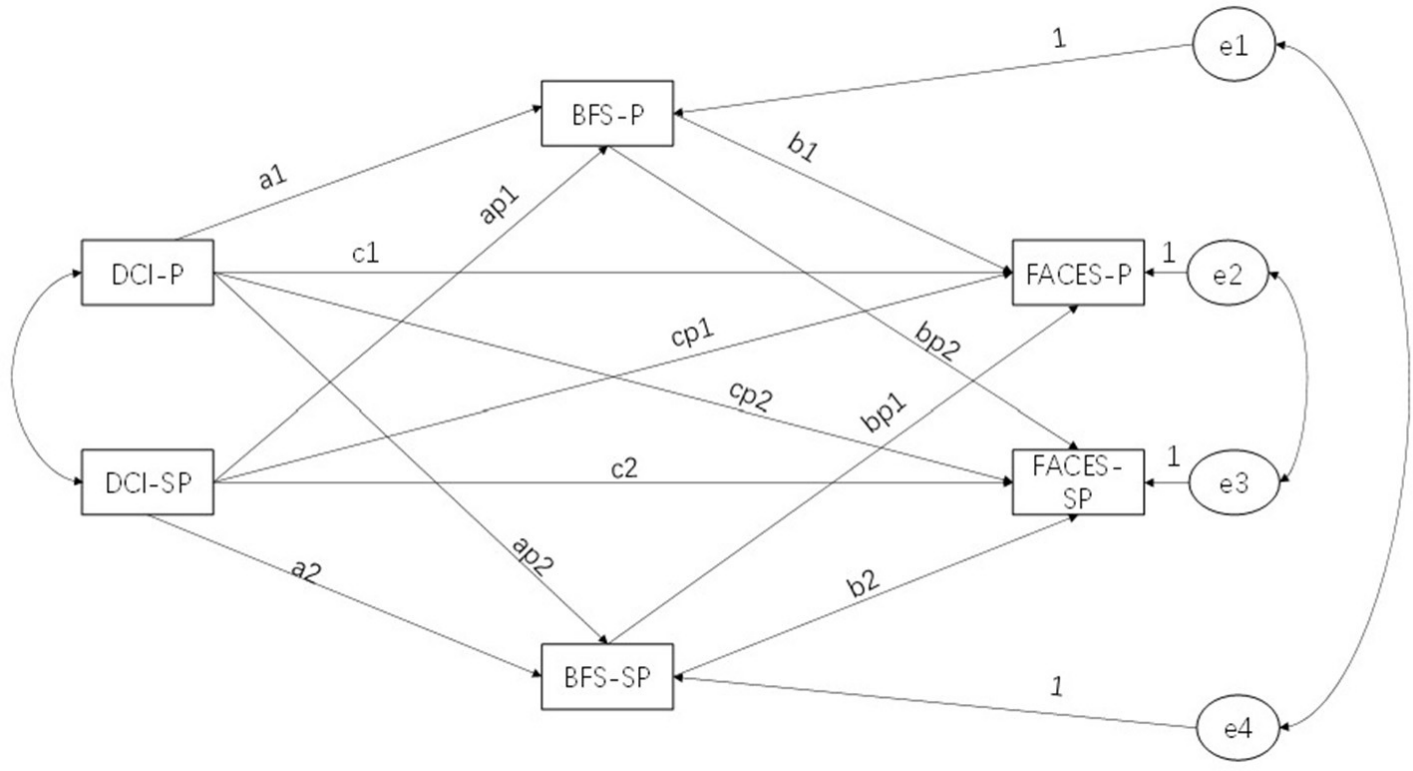
Basic model structure. DCI, Dyadic Coping Inventory; BFS, Benefit Finding Scale; FACES, Family Adaptability and Cohesion Evaluation Scales; *P*, patient; SP, spouse.

This study uses the ABC-X theory and the actor–partner interdependence model for analysis. Reuben Hill, founder of family stress theory, proposed the ABC-X stress theory model in 1949 (25). It is a relatively comprehensive family stress theory model. The model contains four factors: A represents the stressor event; B represents the resources to cope with; C represents the cognition or evaluation of the stressful event; and X represents the outcome, that is, the degree of harm caused by the stress or crisis to the individual. In this study, A is a cancer stress event, B is dyadic coping, C is benefit finding, and X is family adaptation. The actor–partner interdependence model (APIM) is a data analysis method proposed by Kenny and Cook in 1999 that allows researchers to analyze how dependent variables are affected by the predictor variables of another party (the partner effect) as well as their predictor (the actor effect) (26). The actor–partner interdependent mediation model (APIMeM) is an APIM-based model proposed by Kenny and Ledermann, which includes mediation variables (27). This model allows estimation of the mediating effects of benefit finding in patient–spouse dyadic relationships (see Figure 1).

## Materials and methods

### Participants

A cross-sectional survey method was employed in this study. We surveyed breast cancer patients and their spouses who attended breast surgery, radiotherapy, and chemotherapy sessions in three Grade III hospitals in Liaoning Province from April to November 2023 using a convenient sampling method. The inclusion criteria were as follows: (1) age ≥ 20 years and married; (2) pathological diagnosis of breast cancer; (3) the patient must be aware of the diagnosis of her disease; (4) the patient must be able to communicate normally and have no diagnosed mental disorder according to *Diagnostic and Statistical Manual of Mental Disorders*, 5th edition (DSM-5). The exclusion criteria were as follows: (1) widowed or divorced status (patient or spouse); (2) have the intention to withdraw from the survey (patient or spouse); and (3) history of mental problems (patient or spouse).

**Figure 2:**
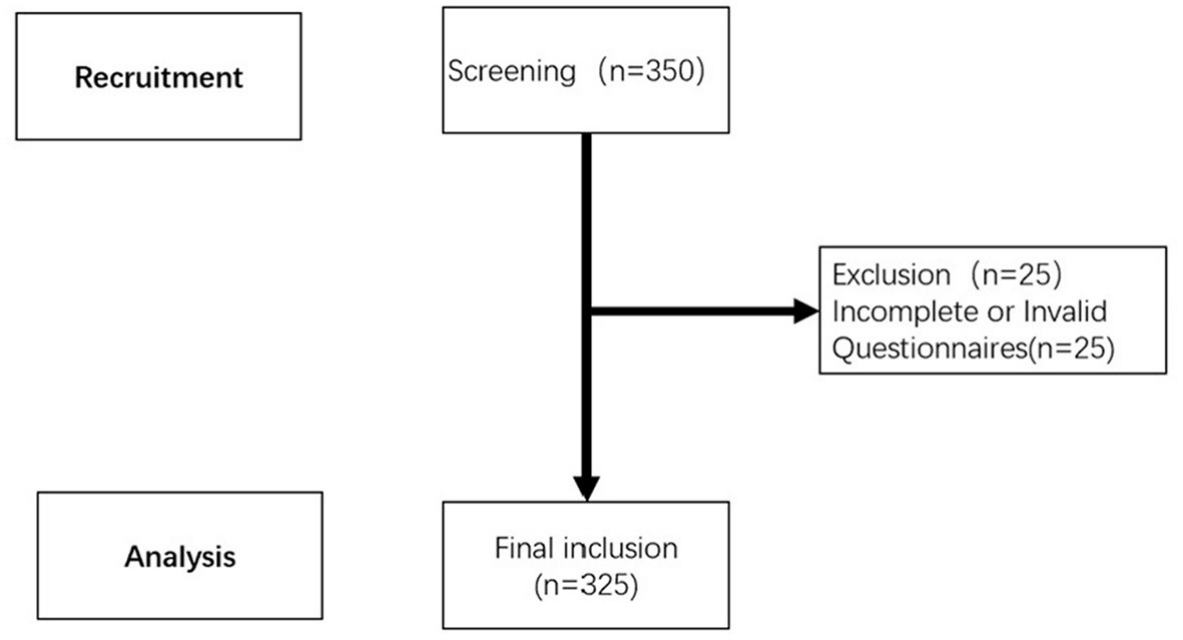
Flow diagram of study participants.

In this study, Gpower3.1 software was used to calculate the sample size. According to the literature review, α value was set to 0.05, the statistical power (1 – β) to 0.90, and the effect size to 0.25 (28). The calculation showed that a minimum of 275 pairs (including patients and spouses) were required, and we collected 325 valid data pairs (see Figure 2).

### Data collection

Before data collection, researchers received uniform training to ensure that the instructions communicated to study subjects remained consistent. The researchers explained the purpose and significance of the study to the subjects and guided them to sign a written informed consent. To ensure data reliability, the subjects were instructed to complete the survey in two different locations and were not allowed to discuss it with one another. At the same time, the researchers made sure to be always available to help study subjects who had difficulty reading, writing, or understanding the survey. Immediately after the survey was completed, the questionnaires were collected and checked to ensure that no missing items were present and that invalid questionnaires were removed. To protect the privacy of research subjects, all information was anonymized. The research followed the ethical principles of the Declaration of Helsinki and was approved by the Ethics Committee of Jinzhou Medical University (approval number: JZMULL2023026).

### Measurements

#### Dyadic coping inventory

DCI was developed by Bodenmann (29) in 2008 to assess how effectively couples cope with stress. In 2016, Chinese scholar Xu et al. (30) translated the DCI scale into Chinese. The scale consists of 37 items divided into five dimensions using a 5-point Likert score with an overall range of 35–175 points. A score below 111 indicates a low level of binary coping, a score between 111 and 145 represents a moderate level, and a score above 145 indicates a high level of binary coping, with a higher score representing a better ability to cope. In this study, the value of Cronbach's α was 0.942 for the patient and 0.916 for the spouse.

#### Benefit finding scale

BFS was originally compiled by Antoni et al. (31) in 2001 for breast cancer patients. The higher the total score, the greater the level of benefit finding. In this study, the Chinese version of the Benefit Finding Scale adapted by Liu et al. (32) was used to assess the level of benefit finding in breast cancer patients. The scale contains 22 items covering six dimensions and uses a 5-point Likert score, with a higher score indicating greater benefit finding. In this study, the value of Cronbach's α was 0.923 for patients and 0.889 for spouses.

#### Family Adaptability and Cohesion Evaluation Scales

FACES is the most commonly used family adaptation scale. FACES was developed by Olson et al. (33) in 1979. Chinese scholars translated the FACES scale into Chinese. They revised it to form the Chinese version of the FACES II-CV scale (34), which contains 30 items divided into two subscales: family intimacy and family adaptability. The intimacy subscale includes 16 items, while adaptability contains 14 items, with each item scored on a five-point Likert scale. The content of the family adaptability dimension is more appropriate, as it better reflects the individual's satisfaction with family adaptation. In this study, the value of Cronbach's α was 0.957 for the patient and 0.965 for the spouse.

### Sociodemographic and clinical characteristics

The demographic information of patients and spouses was collected using self-made questionnaires. At the same time, clinical characteristics of patients and their spouses were obtained through medical record review. All the contents of our investigation were approved by the patients and patients provided their informed consent.

### Quality assurance

Under the tutor's guidance, we edited and standardized the questionnaire and two members of the research team were selected to administer the questionnaire. The study group members distributed questionnaires to protect patients' privacy and encourage their active participation. The responses were put through a double-input and verified using Excel software to ensure the effectiveness and accuracy of the questionnaire.

### Statistical analysis

Descriptive statistical methods, including frequency distribution, mean, and standard deviation, were used to summarize demographic characteristics, cancer-related factors, family adaptation, benefit finding, and dyadic coping. We used the paired-sample *t*-test to describe the differences between patients and spouses regarding the primary study variables, and Pearson's correlation analysis was performed to describe the associations between patients and spouses on the key variables. In this study, we treated patients and spouses as distinguishable data sets. Using APIMeM, we studied the effect of dyadic coping between patients and their spouses on family adaptation and the mediating role of benefit finding. The APIMeM (27, 35) was used to test the hypotheses using bootstrap analysis with 5,000 bootstrap samples and a 95% confidence interval. The dyadic model was verified using χ^2^/df (value < 3), comparative fit index (CFI value ≥ 0.90), Tucker–Lewis index (TLI value ≥ 0.90), and root mean square error of approximation (RMSEA value ≤ 0.08). SPSS25.0 and AMOS24.0 were used for conducting statistical analysis. A *P*-value of <0.05 was considered statistically significant.

## Results

The demographic characteristics of breast cancer patients and their spouses showed that the majority of participants were office workers (51.6% of patients and 50.8% of spouses). Regarding marital status, the majority were married for the first time (77.2% of patients and 86.2% of spouses). Individual monthly income was predominantly in the range of 8,000 yuan and above (39.3% of patients and 45.5% of spouses). Regarding education, the majority of them had attained at least a primary or middle school level (64.3% of patients and 42.5% of spouses). In the majority of families with breast cancer patients, the spouse was in good health, with no health problems, accounting for 82.2%. Additionally, the majority of spouses cared for patients for <1 month (43.1%). In addition, a vast majority of breast cancer patients had a family history of the disease (74.1%), and the main type of breast cancer was *in situ*, accounting for 96.9%. See Table 1 for more detailed data.

**Table 1:** Demographic and cancer-related characteristics (*n* = 325).

**Variable**	**Classification**	**Patient**		**Spouse**	
		**Frequency**	**Percent**	**Frequency**	**Percent**
Occupation	Worker	22	6.7	22	6.77
	Farmer	39	12	51	15.69
	Employee	168	51.6	165	50.77
	Health care workers	37	11.3	41	12.62
	Teacher	39	12	29	8.92
	Retired	20	6.1	17	5.23
Marital status	First marriage	251	77.2	280	86.15
	Remarriage	74	22.7	45	13.85
Place of residence	City	161	49.5		
	Town	125	38.4		
	Countryside	39	12		
Personal monthly income	<1,000	4	1.2		
	1,000–3,000	42	12.9	48	14.77
	3,001–5,000	51	15.6	42	12.92
	5,001–8,000	100	30.7	87	26.77
	>8,000	128	39.3	148	45.54
Education level	Bachelor's degree or above	0	0	11	3.38
	College	38	11.6	95	29.23
	High school	78	24	81	24.92
	Primary school, Middle school	209	64.3	138	42.46
Family history of breast cancer	No	84	25.8		
	Yes	241	74.1		
Have you ever been diagnosed with other breast diseases?	No	6	1.8		
	Yes	319	98.1		
Your type of breast cancer	Carcinoma *in situ*	315	96.9		
	Invasive cancer	10	3.07		
The clinical classification of your breast cancer	I	204	62.7		
	II	110	33.8		
	III	11	3.3		
What kind of breast cancer surgery did you have?	Radical resection + lymph node dissection	157	48.3		
	Modified radical resection + lymph node dissection	98	30.1		
	Breast conservancy	70	21.5		
Relapse or not	Yes	70	21.5		
	No	255	78.4		
Medical payment status	At your own expense	4	1.2		
	Full reimbursement	29	8.9		
	Partial reimbursement	292	89.8		
Your breast cancer site	Left	309	95.08		
	Right	9	2.77		
	Both sides	7	2.15		
You have no health problems	Yes			267	82.15
	No			58	17.85
Have there been any major life stress events in the past 3 months	No			73	22.46
	Yes			252	77.54
How many days have you cared for the patient?	<1 month			140	43.08
	1–3 months			95	29.23
	3–6 months			90	27.69
Have someone with you to help care for the patient?	No			239	73.54
	Yes			86	26.46
Is there anyone else to take care of in addition to the caregiver?	No			186	57.23
	Yes			139	42.77

Table 2 describes the mean value of the variables, the standard deviation, and whether each variable is different in the patient–spouse binary. The paired-sample *T*-test showed significant differences between patients and spouses in dyadic coping, benefit finding, and family adaptation. Patients' binary coping (115.45 ± 15.752), benefit finding (64.18 ± 11.793), and family adaptation (44.93 ± 8.906) were significantly lower than spouses' binary coping (125.32 ± 15.648), benefit finding (70.56 ± 11.941), and family adaptation (46.90 ± 8.973). Table 3 shows the correlation of the study variables. In the correlation analysis, significant associations were found between patient–spouse binary coping (*r* = 0.144, *p* < 0.001), benefit finding (*r* = 0.198, *p* < 0.001), and family adaptation (*r* = 0.525, *p* < 0.001), suggesting that patient–spouse binary relationships were not independent.

**Table 2:** Means, and SDs of the study variables for patient–spouse dyads (*N* = 325 dyads).

		**Means**	**SD**	***P* ^a^**
DCI	Patients	115.45	15.752	*P =* 0.01
	Spouse	125.32	15.648	
BFS	Patients	64.18	11.793	*p* < 0.001
	Spouse	70.56	11.941	
FACES	Patients	44.93	8.906	*p* < 0.001
	Spouse	46.9	8.973	

a Paired-sample *t*-test.

BFS, Benefit Finding Scale; DCI, Dyadic Coping Inventory; FACES, Family Adaptability and Cohesion Evaluation Scales.

**Table 3:** Pearson's correlations and descriptive statistics of the study variables (*n* = 325).

**Variable**	**M**	**SD**	**DCI-sp**	**BFS-sp**	**FACES-sp**	**BFS-p**	**DCI-p**	**DCI-p**
DCI-sp	125.32	15.648	1					
BFS-sp	70.56	11.941	0.394^**^	1				
FACES-sp	46.90	8.973	0.562^**^	0.429^**^	1			
BFS-p	64.18	11.793	0.183^**^	0.198^**^	0.341^**^	1		
DCI-p	115.45	15.752	0.144^**^	0.083	0.341^**^	0.546^**^	1	
DCI-p	44.93	8.906	0.210^**^	0.191^**^	0.525^**^	0.611^**^	0.676^**^	1

BFS, Benefit Finding Scale; DCI, Dyadic Coping Inventory; FACES, Family Adaptability and Cohesion Evaluation Scales; P, patient; SP, spouse.

^**^P < 0.001.

### Actor–partner interdependence mediation model

In this study, we constructed a subjective and actor–partner mediation model of benefit finding, dyadic coping, and family adaptation for breast cancer patients and their spouses. The results showed that the model fit well (CMIN/DF = 2.754, CFI = 0.984, TLI = 0.961, IFI = 0.985, GFI = 0.983, RMSEA = 0.074).

The results in Tables 4, 5 show that the dyadic coping of patients (*B* = 0.276; *P* < 0.05; lower = 0.188; upper = 0.354) and spouses (*B* = 0.244; *P* < 0.05; lower = 0.163; upper = 0.317) impacts family adaptation. In addition, in the actor effect, the dyadic coping of patients (*B* = 0.397; *P* < 0.001; lower = 0.31; upper = 0.477) and spouses (*B* = 0.298; *P* < 0.001; lower = 0.191; upper = 0.402) impacts the benefit finding. In the partner effect, the patient's dyadic coping does not affect the partner's benefit finding (*B* = 0.02; *P* = 0.608), but the spouse's dyadic coping affects the patient's benefit finding (*B* = 0.08; *P* = 0.03; lower = 0.008; upper = 0.153).

**Table 4:** Significant and non-significant direct effects of study variables.

**Parameter**	**Label**	***B***	**Lower**	**Upper**	***P***
BFS-P ← DCI-P	a1	0.397	0.31	0.477	0.000
BFS-SP ← DCI-SP	a2	0.298	0.191	0.402	0.000
BFS-SP ← DCI-P	ap2	0.02	−0.059	0.098	0.608
BFS-P ← DCI-SP	ap1	0.08	0.008	0.153	0.03
FACES-P ← DCI-P	c1	0.276	0.188	0.354	0.001
FACES-SP ← DCI-SP	c2	0.244	0.163	0.317	0.001
FACES-P ← BFS-P	b1	0.243	0.132	0.352	0.000
FACES-SP ← BFS-SP	b2	0.168	0.075	0.271	0.001
FACES-SP ← DCI-P	cp2	0.116	0.053	0.188	0.000
FACES-P ← DCI-SP	cp1	0.031	−0.018	0.078	0.224
FACES-P ← BFS-SP	bp1	0.049	−0.015	0.114	0.115
FACES-SP ← BFS-P	bp2	0.082	−0.014	0.179	0.093

*B*, unstandardized estimate; BFS, Benefit Finding Scale; DCI, Dyadic Coping Inventory; FACES, Family Adaptability and Cohesion Evaluation Scales; P, patient; SP, spouse.

**Table 5:** Bootstrap test for indirect effects for the actor–partner interdependence mediation model with dyadic coping inventory as an independent variable, benefit finding as a mediator, and family adaptability as outcome.

**Effect**		***B***	**Lower**	**Upper**	***P***
**Actor effect (Individual's dyadic coping inventory**→**Individual's family adaptability) Patient**					
Total effect		0.373	0.301	0.433	0.001
Total indirect effects		0.097	0.056	0.145	0.000
Actor–actor	DCI-P → BFC-P → FACES-P	0.096	0.056	0.144	0.000
Partner–partner	DCI -P → BFC-SP → FACES-P	0.001	−0.002	0.009	0.378
Direct effects	DCI -P → FACES-P	0.276	0.188	0.354	0.001
**Spouse**					
Total effect		0.3	0.23	0.366	0.001
Total indirect effects		0.057	0.027	0.1	0.000
Actor–actor	DCI -SP → BFC-SP → FACES-SP	0.05	0.022	0.093	0.000
Partner–partner	DCI -SP → BFC-P → FACES-SP	0.007	0	0.021	0.062
Direct effects	DCI-SP → FACES-SP	0.244	0.163	0.317	0.001
**Partner effect (Individual's dyadic coping inventory**→**Partner's family adaptability) Patient**					
Total effect		0.065	0.014	0.118	0.009
Total indirect effects		0.034	0.011	0.065	0.006
Actor–partner effect	DCI-SP → BFC-SP → FACES-P	0.014	−0.003	0.038	0.095
Partner–actor effect	DCI-SP → BFC-P → FACES-P	0.019	0.003	0.045	0.02
Direct effects	DCI-SP → FACES-P	0.031	−0.018	0.078	0.224
**Spouse**					
Total effect		0.152	0.098	0.21	0.000
Total indirect effects		0.036	−0.001	0.077	0.056
Actor–partner effect	DCI -P → BFC-P → FACES-SP	0.033	−0.004	0.073	0.082
Partner–actor effect	DCI -P → BFC-SP → FACES-SP	0.003	−0.008	0.022	0.539
Direct effects	DCI -P → FACES-SP	0.116	0.053	0.188	0.000

*B*, unstandardized estimate; β, standardized estimate; BFS, Benefit Finding Scale; DCI, Dyadic Coping Inventory; FACES, Family Adaptability and Cohesion Evaluation Scales; P, patient; SP, spouse.

**Figure 3:**
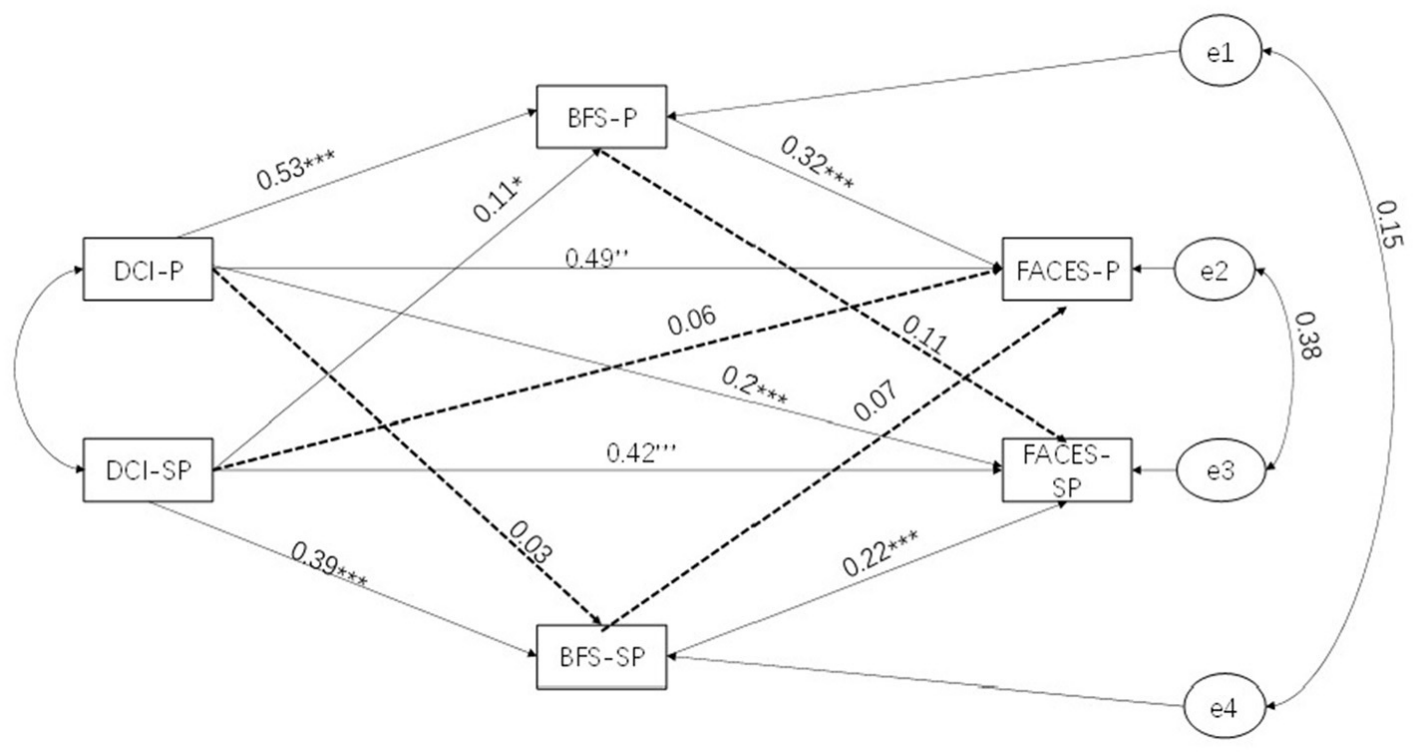
Actor–partner interdependence mediation model. DCI, Dyadic Coping Inventory; BFS, Benefit Finding Scale; FACES, Family Adaptability and Cohesion Evaluation Scales; *P*, patient; SP, spouse; ***p < 0.001, **p < 0.01, *p < 0.05.

In families of breast cancer patients, we have shown that benefit finding plays a mediating role between couples' dyadic coping and family adaptation. Specifically, we observe that the dyadic coping of patients (*B* = 0.096, *P* < 0.001; *B* = 0.05, *P* < 0.001) and their spouses (*B* = 0.050, 95% CI = 0.022–0.093, *P* < 0.001) has an actor effect on family adaptation and that this effect is partly mediated by benefit finding. In other words, the higher the level of dyadic coping of patients and their spouses, the more significant their benefit finding, leading to better family adaptation. In addition, we also found a partner effect: the dyadic coping of spouses can affect the patient's benefit finding and then affect the patient's family adaptation (*B* = 0.019, 95% CI = 0.003–0.045, *P* < 0.05). However, spouses' dyadic coping did not directly affect patients' family adaptation, possibly because patient benefit finding plays a mediating role (see Figure 3).

The percentage of explanatory variation for the benefit finding in patients and spouses was 30.89 and 15.6%, respectively. The percentage of explanatory variation for family adaptation in patients and spouses was 55.03 and 44.05%, respectively.

## Discussion

Previous studies have focused on the factors influencing family adaptation (36, 37) and the impact of coping styles on family adaptation (38). This study further expands on these frameworks, exploring the actor–partner effect of dyadic coping on family adaptation from a dyadic perspective and examining the mediating role of benefit finding in this effect. This is the first study to examine the effects of binary coping and benefit finding on family adaptation.

In this study, the positive dyadic coping of patients and spouses can directly affect their family adaptation. Previous studies have shown that good dyadic coping between couples can promote effective communication, enhance emotional stability, and contribute to family harmony (39). Effective dyadic coping enables couples to think from each other's perspective and understand one another when facing challenges, thereby enhancing family stability (40). For patients, the positive support, companionship, and care provided by the spouse can reduce the emotional pressure on the patient, convey love and care, and improve the confidence to overcome the disease and find the beauty of life (41). For spouses, good dyadic coping is considered an intrinsic resource for managing family crisis, which helps reduce caregivers' stress and burden (42, 43). This allows the spouse to work with the patient to overcome the challenges of the disease and have a positive experience.

A high level of dyadic coping is key for families to survive the crisis smoothly. The latest study confirms the conclusions of previous studies that the coping styles of patients and spouses impact the benefits finding (44, 45). When coping becomes a support system, good dyadic coping allows patients to trust that they will have support within the family when they encounter challenges (29). This support helps to enhance the individual's ability to overcome difficulties and thus find benefits in life. In addition, our findings show that the degree of benefit finding in patients and spouses can predict their family adaptation. Previous research has shown that individuals with higher levels of benefit finding are more inclined to cope with illness positively (46). Benefit finding helps individuals alleviate anxiety and depression while enhancing their ability to overcome difficulties so that families can better cope with crises and show good adaptability (41). Our study also found that the dyadic coping of breast cancer patients and their spouses indirectly affects their family finding through benefit findings. In the presence of negative emotions such as pessimism, good dyadic coping can be used as a resource within the family to help cope with crises and maintain family stability (47).

## Limitations

There are some limitations to this study. First, it is impossible to infer a causal relationship between benefit finding, dyadic coping, and family adaptation using a cross-sectional design. At the same time, there is no restriction on the timing of the cancer diagnosis, which may lead to confounding factors affecting the outcome. Future longitudinal study designs are recommended to explore the relationship between these variables in depth and to understand the mechanisms by which patients at different stages of cancer interact with their spouses. Second, the data in this study are based on self-reports and there is a risk of reporting bias. The couples involved in the study may have had good family relationships, limiting the generalizability of the results. Future studies may consider using other data collection methods to explore couples with a less positive family atmosphere. Third, the study focused solely on breast cancer in women and did not take into account gender differences, other types of cancer, or the racial demographics of the population. Therefore, it is not possible to explore in depth the differences between various cancer types and the differences between various ethnic groups. Future studies are suggested to provide a more specific analysis of different cancer types and to consider the effects of gender and ethnicity. Finally, the majority of spouses in our study cared for patients for a shorter period of time and their coping styles changed over time (some spouses had better coping styles at first, but these changed as they became bored or disappointed during the course of caring). In addition, the duration of a breast cancer patient's marriage to their spouse is also a factor to consider. Therefore, in future studies, we need to conduct longitudinal studies to explore how dyadic coping, benefit finding, and family adaptation evolve during the course of patient care.

## Conclusion

This study highlights a codependent relationship between breast cancer patients and their spouses. Dyadic coping in spouses is associated with beneficial findings for patients and further influences the adaptation of patients' families. Therefore, clinical staff can use the Dyadic Coping Scale to identify patients and spouses with poor coping abilities and provide positive psychological interventions to enhance the dyadic coping ability between couples to help them overcome the problems encountered during treatment and better cope with family crises. At the same time, clinicians should encourage the patient's spouse to participate actively in the cancer treatment process to help the couple develop good coping abilities. Through combined psychological interventions, communication and understanding between cancer couples can be improved. This, in turn, helps them find good things in life, cope more effectively with family crises, and improve family adaptation. In implementing this measure, clinicians should provide comprehensive support and education for patients and their spouses, along with timely and effective adjustments of intervention strategies to prevent placing unnecessary burden on the patients' families. Encouraging patients and their spouses to participate in developing and implementing the care plan can improve communication and understanding between couples, strengthen their ability to cope with the crisis, and help the entire family better adapt to the crisis.

## Data Availability

The raw data supporting the conclusions of this article will be made available by the authors, without undue reservation.
